# Biotechnological Uses of Archaeal Proteins

**DOI:** 10.1155/2015/809758

**Published:** 2015-10-12

**Authors:** Frédéric Pecorari, Vickery L. Arcus, Juergen Wiegel

**Affiliations:** ^1^Cancer Research Center of Nantes-Angers, INSERM UMR 892, CNRS UMR 6299, University of Nantes, 44007 Nantes, France; ^2^School of Science, University of Waikato, Hamilton, New Zealand; ^3^Departments of Microbiology and of Biochemistry & Molecular Biology, University of Georgia, Athens, GA 30605, USA

Many industrial/biotechnological processes take place under extreme conditions of temperature, pH, salinity, or pressure which are not suitable for activities of proteins from model eukaryotic or common neutrophilic, mesophilic, and prokaryotic microorganisms. In contrast, Archaea offer a large panel of extremophile organisms that express proteins that are able to remain properly folded and functional under the harshest biophysical conditions.

The study of this group of organisms has uncovered archaeal enzymes and proteins with unusual properties compared to their traditional homologues. In addition, with their ease of production and better-behaved samples for X-ray crystallography, for example, archaeal proteins are often more convenient for structural biology studies than their eukaryotic equivalents. The knowledge thus gained can open routes to commercial biotechnological applications. These last years, with the emergence of next generation sequencing techniques to decode whole genomes and metagenomes and the pressure to develop “greener” industrial processes, the rate of new archaeal proteins reported has significantly increased, thereby widening again their potential of applications. In this special issue of Archaea, we present selected papers dealing with the uses of archaeal proteins as tools for various fields of biotechnologies and research.

DNA and RNA ligases are essential enzymes in living cells and have applications in molecular biology. A review by M. Tanabe et al. discusses the uses of DNA ligases and recent progress in deciphering their catalytic mechanisms* via* structural studies, and they describe how protein engineering can improve ligation efficiency of an archaeal DNA ligase over a broad temperature range. In another paper on ligases, C. R. Chambers and W. M. Patrick present the current state of knowledge on archaeal nucleic acid ligases including RNA ligases, highlighting their remarkable properties relevant to biotechnologists, and they discuss the modifications of the activities of archaeal RNA ligases by directed mutagenesis to develop more efficient molecular biology protocols.

J. A. Littlechild reviews research regarding the discovery and potential applications of a range of thermophilic archaeal proteins, illustrating the power of archaeal enzymes for various industrial biocatalysis. Then, an article by V. M. Gumerov et al. describes the characterization of a novel thermostable and multifunctional *β*-glycosidase from* Acidilobus saccharovorans* that displays a high tolerance to glucose, a desired property for such enzymes used to process lignocellulose biomass. C.-H. Wu et al. present a review summarizing the strategies used in engineering and characterizing three different forms of soluble hydrogenase I from the hyperthermophile* Pyrococcus furiosus*, an enzyme which has been used* in vitro* for hydrogen production.

Archaea are not only interesting for catalysis applications. J. C. Charlesworth and B. P. Burns give a comprehensive overview of archaeal low-molecular weight compounds including peptides with antimicrobial properties which can be exploited for biotechnological purposes. Finally, in their review, J. M. Miller and E. J. Enemark exemplify with MCM helicases how crystallography of archaeal homologues can be helpful to decipher structure/function relationships of their eukaryotic versions which are more difficult to crystallize.

We hope this special issue will provide the reader with an up-to-date overview of some of the diverse applications of archaeal proteins and, perhaps, prompt further research in this largely untapped field with rich prospects for the future of many biotechnological applications.

*Frédéric Pecorari* *Vickery L. Arcus* *Juergen Wiegel*

